# Supplementary material for: Nucleolin-Sle A Glycoforms as E-Selectin Ligands and Potentially Targetable Biomarkers at the Cell Surface of Gastric Cancer Cells
Source: Cancers (Basel). 2020 Apr 2;12(4):861. doi: 10.3390/cancers12040861 (PMC7226152; doi:10.3390/cancers12040861)
Supplement: Supplementary file 1 [file cancers-12-00861-s001.pdf]

Supplementary Materials: Nucleolin-SLeA glycoforms as E-selectin ligands and potentially targetable biomarkers at the cell surface of gastric cancer cells

Elisabete Fernandes, Rui Freitas, Dylan Ferreira, Janine Soares, Rita Azevedo, Cristiana Gaiteiro[a], Andreia Peixoto, Sofia Cotton, Marta Relvas-Santos, Luis Pedro Afonso, Carlos Palmeira, Maria José Oliveira, Rita Ferreira, André M. N. Silva, Lúcio Lara Santos, José Alexandre Ferreira

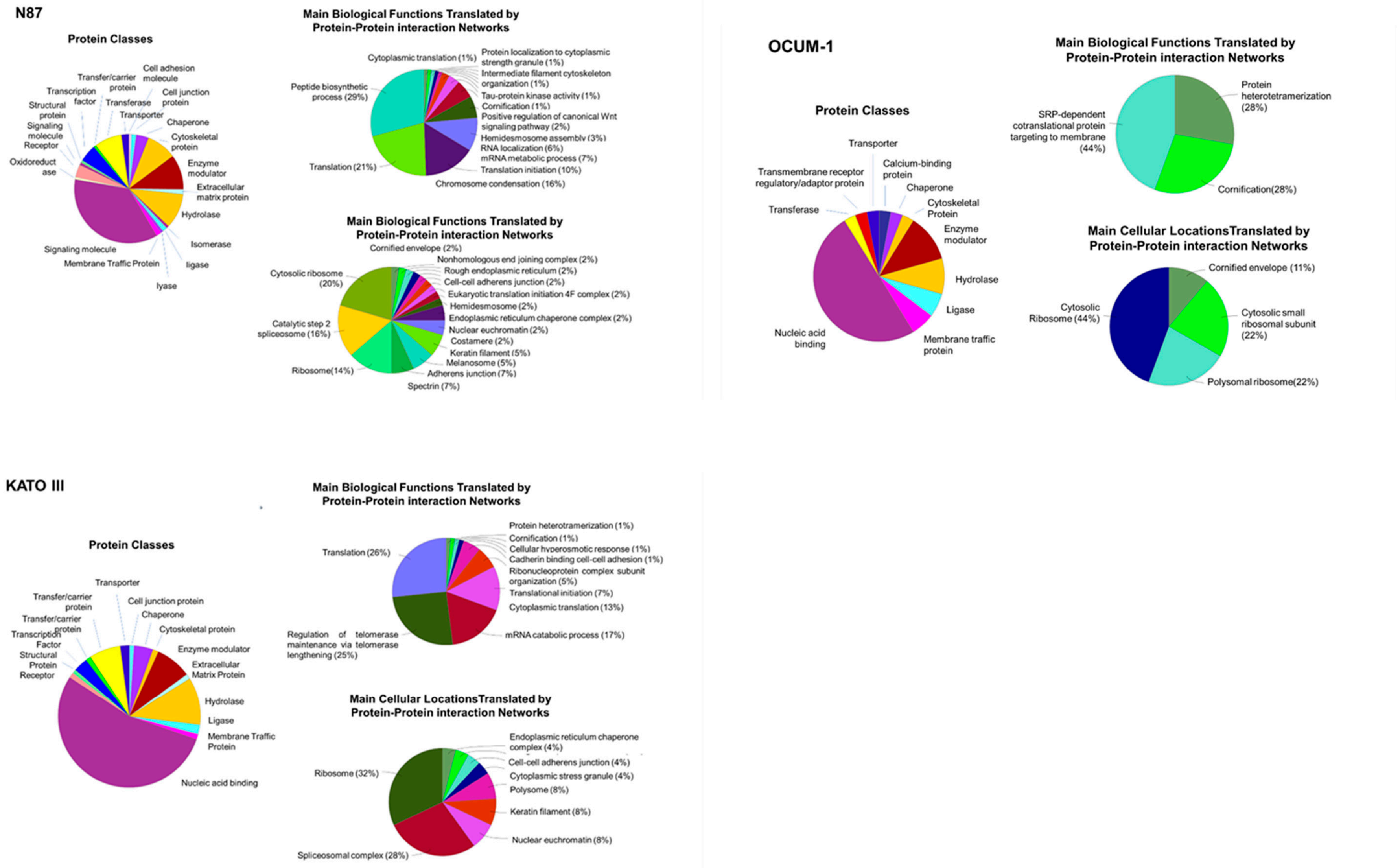

Figure S1. Main protein classes and associated biological functions translated by protein-protein interactions and main subcellular localization for glycoproteins expressing O-glycosylated glycans with SLe^{A} and showing affinity for E-selecting for N87, OCUM-1 and Kato III cell lines. A wide number of different protein classes are represented for each cell line (20 for N87, 11 for OCUM-1, 14 for KATO III), denoting significant differences

between the cell models. Nevertheless, the main represented protein classes included signaling and nucleic acid binding for N87 and OCUM-1 and N87 cell lines, respectively. Strikingly, many of these glycoproteins (20-60%) were primarily associated with protein translation and biosynthesis in the ribosomes, spliceosome complexes and even at the nuclear euchromatin. Proteins typically found in the cytoplasm and the cytoskeleton were also detected (10-20%). On the other hand, only 10-15% of them could be associated to the plasma membrane, cell junctions and the extracellular space, known to typically undergo O-glycosylation across the secretory pathways.

[1]MVKLAKAGKNQGDPKKMAPPPKEVEED**S**EDEEM**S**EDEEDDSSGEEVVIPQ

KKGKKAAA**TS**AKKVVV**S**P**T**KKVAVA**T**PAKKAAV**T**PGKKAAA**T**PAKK**T**

V**T**PAKAV**TT**PGKKGA**T**PGKALVA**T**PGKKGAAIPAKGAKNGKNAKKED**S**

DEEEDDDSEEDEEDDEDEDEDEDEDEIEPAAMKAAAAAPASEDEDDEDDEDDEDD

DDDEEDDSEEEAME**TT**PAKGKKAAKVVPVKAKNVAEDEDEEEEDDEDEDDDD

DEDDEDDDDEDDEEEEEEEEEEEPVKEAPGKRKKEMAKQKAAPEAKKQKVEG**T**

EP**T**TAFNLFVGNLNF**N**KSAPELKTGISDVFAKNDLAVVDVRIGMTRKFGYVDF

ESAEDLEKALELTGLKVFGNEIKLEKPKGKDSKKERDARTLLAKNLPYKVTQDEL

KEVFEDAAEIRLVSKDGKSKGIAYIEFKTEADAEKTFEEKQGTEIDGRSISLYYTGE

KGQNQDYRGGK**NS**TWSGESKTLVLS**N**LSYSATEETLQEVFEKATFIKVPQN

QNGKSKGYAFIEFASFEDAKEALNSCNKREIEGRAIRLELQGPRG**S**PNAR**S**QP

SKTLFVKGLSEDTTEETLKESFDGSVRARIVTDRETGSSKGFGFVDFNSEEDAKA

AKEAMEDGEIDGNKVTLDWAKPKGEGGFGGRGGGRGGFGGRGGGRGGRGG

FGGRGRGGFGGRGGFRGGRGGGGDHKPQGKKTKFE[710]

Figure S2. Potential *N*- (Blue) and *O*-glycosylated (Red) sites for NCL predicted with NetNGlyc 1.0 and NetOGlyc 4.0 bioinformatics tools and using the protein canonical form as reference. The Figure highlights the higher density of potential *O*-glycosylation sites (n=23) in comparison to N-glycosylation sites (*n* = 3).

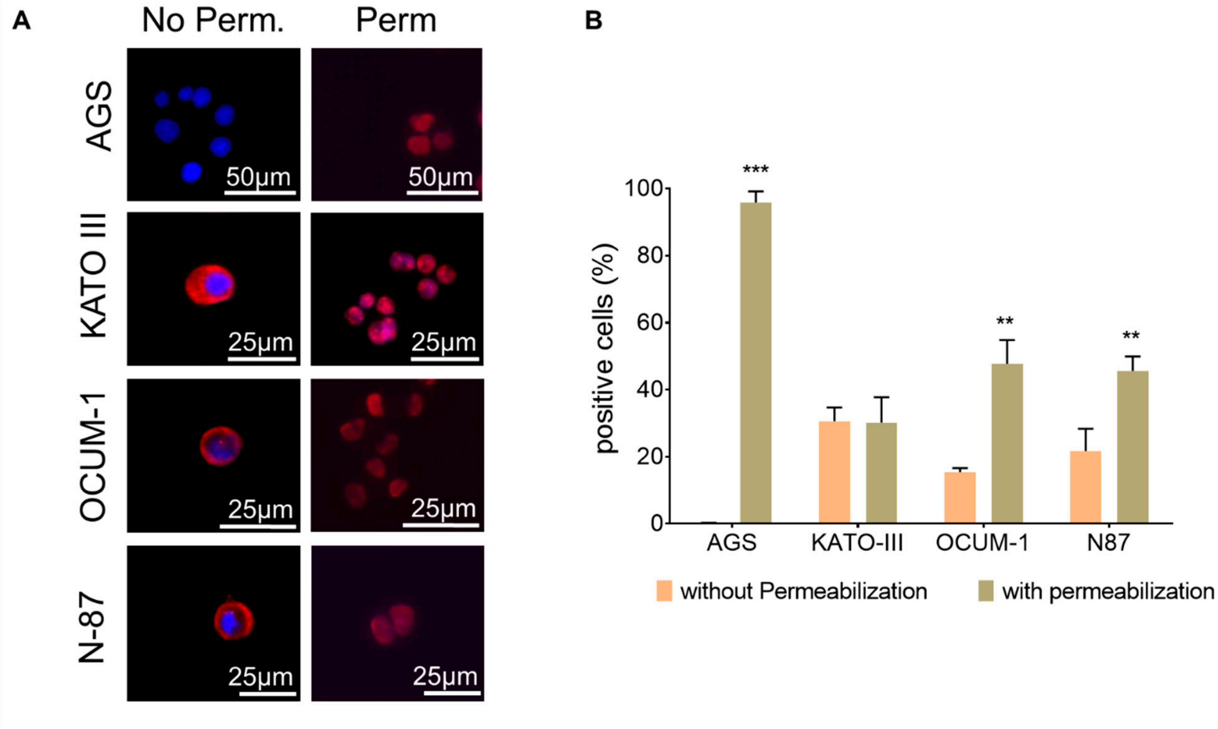

Figure S3. (**A**) Immunofluorescence for NCL, (**B**) Percentage of NCL positive cells and (**C**) NCL levels before and after plasma membrane permeabilization**.** Collectively, this data supports that SLe^{A}-negative AGS cells do not express NCL at the cell membrane, as translated by the absence of NCL expression at the cell membrane before cell membrane permeabilization. Notably, almost all AGS cells expressed intracellular NCL. The SLe^{A} positive cell lines (KATO-III, OCUM-1, N-87) all showed NCL at the cell membrane before permeabilization. After permeabilization the bulk of the staining was found in the nucleus. Notably, the percentage of NCL positive cells for these models varied between 15-30% and significantly doubled for OCUM-1 and N87 cell lines after permeabilization. These observations suggest that these cell lines may present both intracellular and membrane NCL.

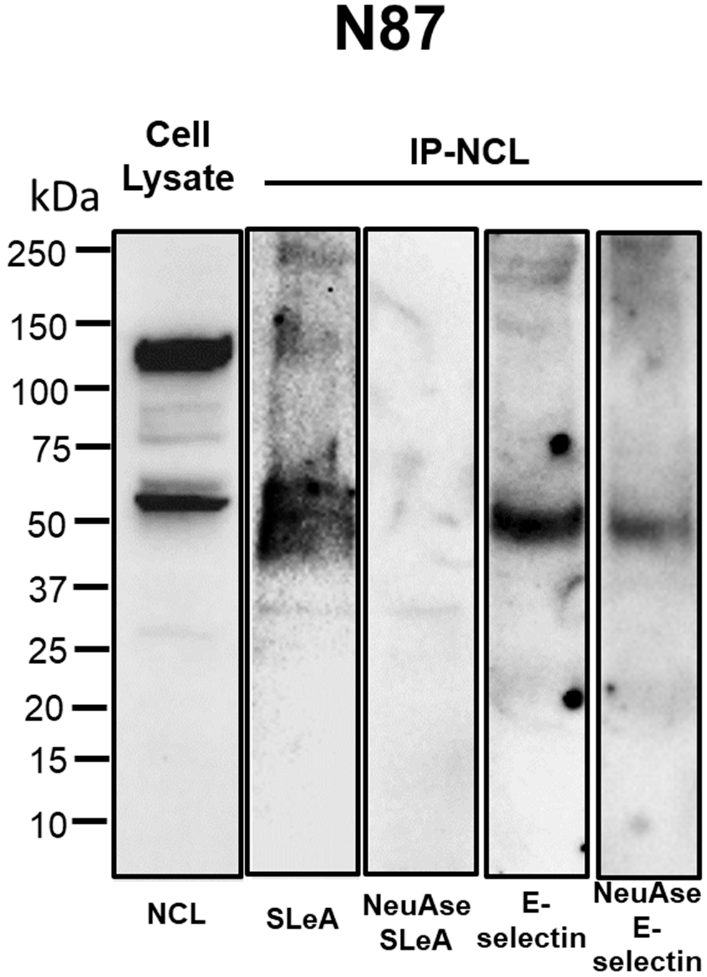

Figure S4. Western blot for NCL in whole cell lysates and SLeA and E-selectin in NCL-IPs. Proteins digested in situ with neuraminidase (NeuAse) where used as negative controls. Collectively these blots support that existence of NCL-SLe^{A} expressing glycoproteins at 100 kDa and bellow, with a major band just above 50 kDa. This band and maybe others of less intensity at higher and lower molecular weights, showed affinity for E-selectin mediated by sialylation. The disappearance and/or reduction the signals after NeuAse digestion supports these observations.

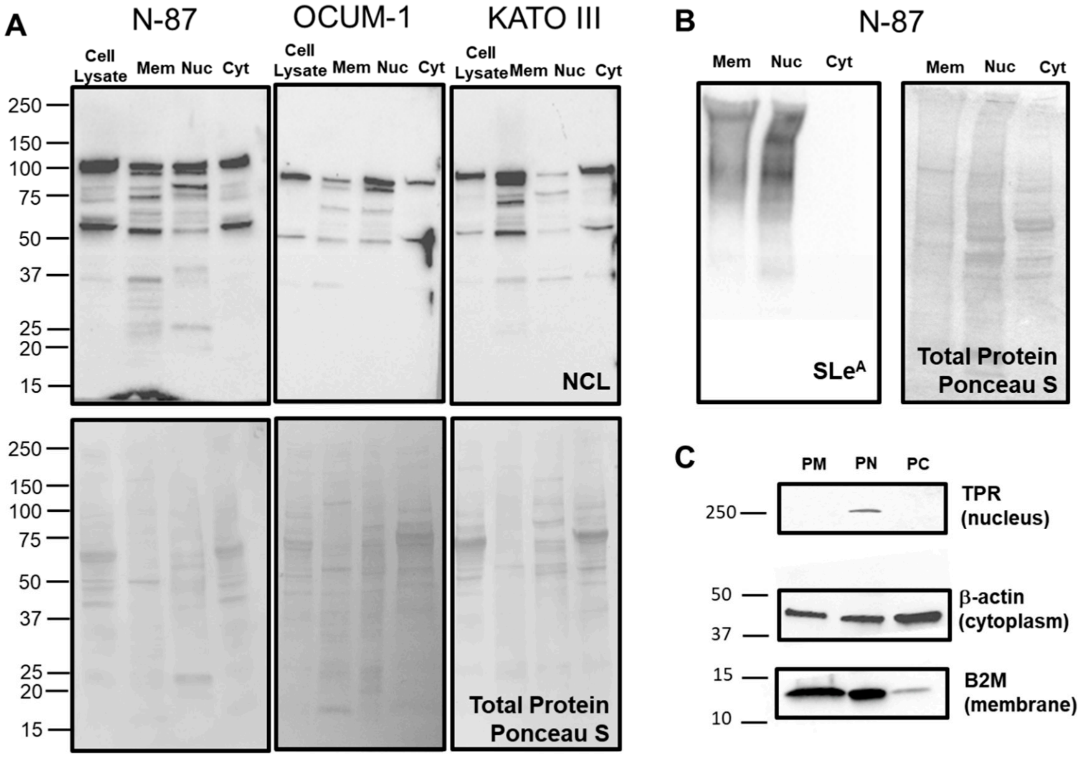

Figure 5. (**A**) NCL expression profiles in the whole cell lysates and different protein extracts enriched for membrane, nuclear and cytoplasmatic proteins. (**B**) SLe^{A} expression in the different fractions. (**C**) Expression of nuclear (TPR), cytoplasm (β-actin) and B2M (plasma membrane) biomarkers across the different fractions for the N87 cell line. Collectively, the panels show the presence of NCL in all subcellular fractions across the three cell lines, with very similar patterns between different cell lines (panel A). The NCL/Total protein (estimated by Ponceau S) ratio suggested that the membrane protein fraction is more enriched for NCL compared to the other two. In addition, all fractions presented bands at approximately 50 kDa and above 100 kDa, suggesting that these proteoforms could be present in all subcellular locations. Nevertheless, NCL expression patterns varied according to the subcellular fraction, suggesting distinct molecular signatures. Notably, the plasma membrane fraction was significantly enriched for SLe^{A} and B2M typically found in at the cell surface (panel B), even though with some contamination with β-actin, suggesting also the presence of cytoplasmatic proteins. However, it did not present TPR, demonstrating little or no contamination with nuclear proteins and reinforcing the membrane origin of some of the observed NCL proteoforms. The nuclear fraction presented high amounts of TPR and, to less extent β-actin. Strikingly, it was also highly contaminated with membrane glycoproteins (SLe^{Ahigh}, $B2M^{high}$), not allowing to fully disclose nuclear-specific signatures. However, the cytoplasmatic fraction suggested little contamination with proteins from other subcellular locations ($SLe^{Anegative}$, $B2M^{low}$, $TPR^{negative}$, β-$actin^{high}$). Taken together, these findings support that NCL at 50 and 100 kDa were not membrane specific. It also strongly suggested the existence of NCL specific proteoforms at the cell membrane, distinct from those of intracellular nature.

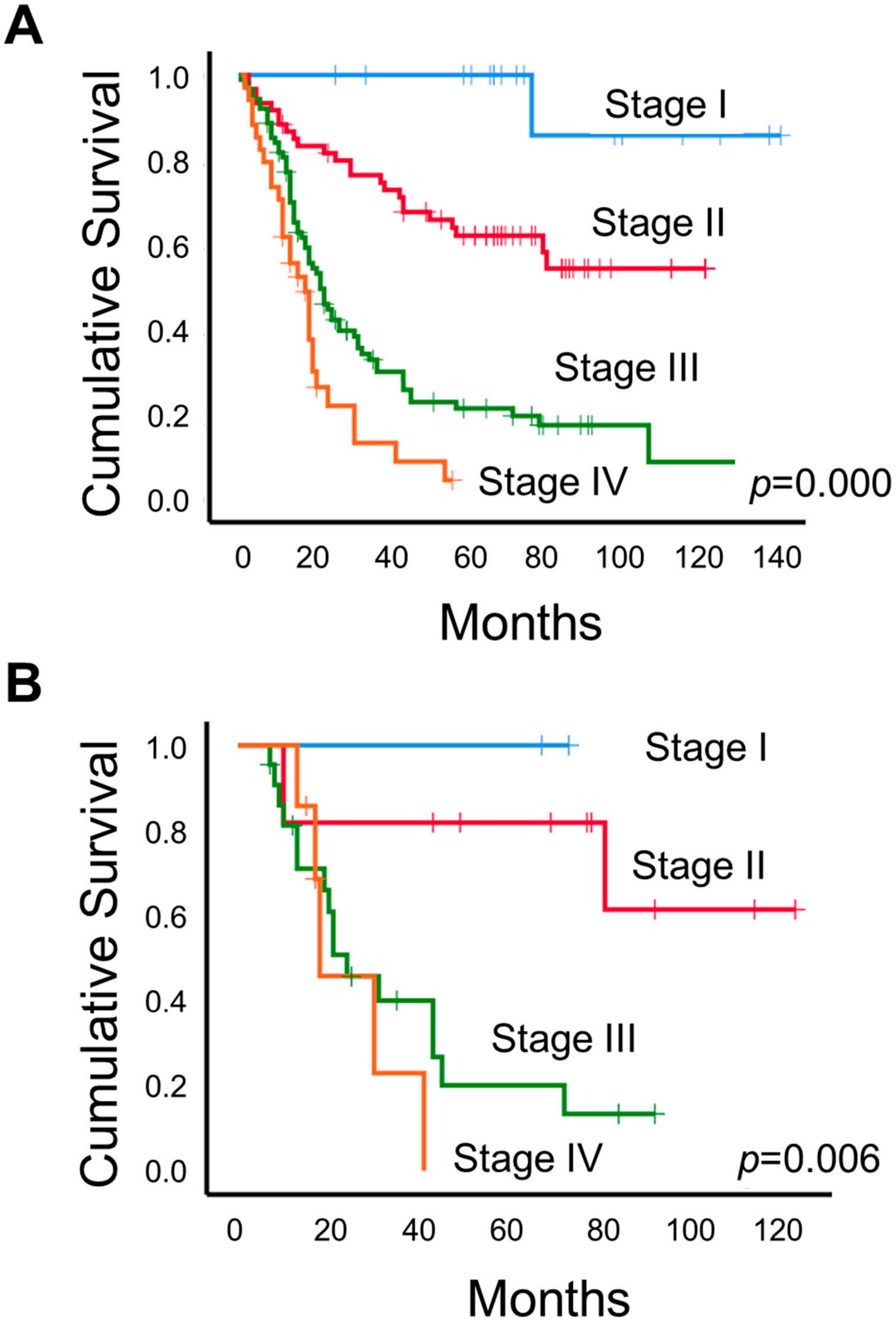

Figure S6. kaplan meier survival curves for the patients' cohort used for A) SLe^{A} and the patient's subset used for B) NCL and NCL-SLe^{A} evaluations. The two datasets reflect a statistically significant decrease in patient's survival with increasing stage of the tumour. The *p*-values in survival analyses reflect the Breslow test.

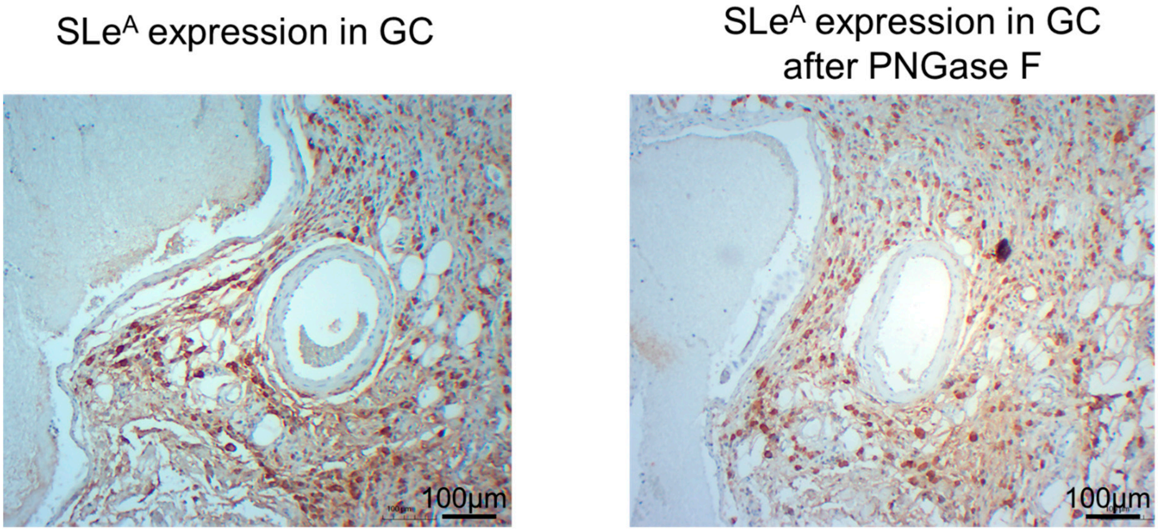

Figure S7. SLe^A expression a gastric tumour section before and after PNGase F digestion. The panels highlight that SLe^A expression in gastric tumours is not significantly affected by PNGase F digestion. Notably, PNGase F-digested bladder tumours (positive control for *N*-glycans expressing SLe^A) showed a strong decrease in SLe^A expression, highlighting that the enzyme is effective in removing *N*-glycans. Moreover, SLe^A expression decreased after neuraminidase digestion (negative control; not shown). These observations support that the bulk of SLe^A expression in gastric tumours derives from *O*-glycans.

Table 1. Origin and clinicopathological features for the GC cell lines adopted for this study.

Cell line	Cell origin	Gender	Age	Lauren classification	Differentiation	Ethnicity
AGS	Primary tumour	Female	54	Intestinal type	Moderately differentiated adenocarcinoma	Caucasian
KATO III	Pleural effusion	Male	55	Diffuse type	N/A	Asian
MKN-74	Liver metastasis	Male	37	Intestinal type	Moderately differentiated tubular adenocarcinoma	Asian
NCI-N87	Liver metastasis	Male	N/A	Intestinal type	Well differentiated carcinoma	N/A
OCUM-1	Primary tumour	Female	38	Diffuse type	Poorly differentiated adenocarcinoma containing signet ring cells	Asian
